# Editorial

**Published:** 1903-12-15

**Authors:** 


### EDITORIAL.

With this issue we conclude the fifty-seventh volume of the DENTAL REGISTER. As we look back over the year we can see many places where we could have improved our work, and yet we congratulate ourselves that we have in so large a measure fulfilled the scope of our forecast at the beginning of the year. Of course we intend to do better things during the coming year, and have already in hand some features which we hope to gradually work into the new volume. We shall continue to record the doings of our local societies, and extract the good things which appear in other periodic literature. The past year brought to us several material advances in a professional way. The one of most notice was, perhaps, the extension of the college course. It took from us also several conspicuous persons who have made their marks on our professional development, by the concentration of their talents and the devotion of their lives to its higher spheres. What the coming year may bring to us, we can not predict; but we shall all be interested to know of it and to help it along, each in his own way. How can we better do this than by keeping in touch with the printed records, and by a close identification with those organizations which have done more to develop our literature and advance us in the science and art of our calling?

It is fair to suppose that in this day of the inexpensive dental periodicals there are more readers of dental literature, than ever before. And it is likely that because of this reading more men will become contributors to dental literature through the dental societies and their printed records. If the recording of these transactions is intelligently and faithfully done the probabilities are that there is a feast of good things in store for us this coming year. Every dentist should provide himself with as many dental journals as he can properly read and assimilate. From three to five dollars carefully spent ought to keep a dentist fairly well in touch with what is transpiring in the world of his calling.

There are all classes of journals, each one having a peculiarity of its own to commend it. If we were asked to select three journals that would serve to keep one fairly-well posted, we should advise first that one journal should be taken which gives itself largely to the publication of important scientific work. Secondly, we should advise a journal given largely to the technical side of our work. As a third we should select a journal that aimed to record the work of such societies as one was most interested in, or at least those meeting in the locality where one should, if he does not, attend and participate in their proceedings. Every progressive and reasonably-successful practitioner should afford himself so much reading; and many ought to go much farther and add other journals of value. This of course should be supplemented by collateral and general reading which increases one's knowledge and culture, and keeps us from becoming narrow and selfish. We may pride ourselves that because of the advances in educational methods and standards by the colleges and State Examining Boards that our calling is destined to jump quickly to one of the higher places, as a profession. But what use will it be if college graduates drop their literary acquirements as soon as they become registered for practice, for the more remunerative technical affairs? Colleges and State Boards can not drag the profession upward in spite of itself. The truth is that these institutions can only reflect the advancement made by the rank and file of the busy and thoughtful practitioners. Haven't we each one an obligation? We know that many realize it; but a vast army seems indifferent, and this is not as it should be.

